# Seminoma and dysgerminoma: evidence for alignment of clinical trials and de-escalation of systemic chemotherapy

**DOI:** 10.3389/fonc.2023.1271647

**Published:** 2023-10-25

**Authors:** Georgina E. Wood, Christopher P. Bunting, Mesel Veli, Rupali Arora, Daniel M. Berney, Constantine Alifrangis, Nicola D. MacDonald, Rowan E. Miller, Jonathan Shamash, Sara Stoneham, Michelle Lockley

**Affiliations:** ^1^ Medical Oncology, University College London Hospital, London, United Kingdom; ^2^ Faculty of Medicine and Dentistry, Queen Mary University of London, London, United Kingdom; ^3^ Histopathology, University College London Hospital, London, United Kingdom; ^4^ Histopathology, Barts Health NHS Trust, London, United Kingdom; ^5^ Department of Gynaecology, University College London Hospital, London, United Kingdom; ^6^ Medical Oncology, Barts Health NHS Trust, London, United Kingdom; ^7^ Paediatric Oncology, University College London Hospital, London, United Kingdom; ^8^ Centre for Cancer Genomics and Computational Biology, Barts Cancer Institute, Queen Mary University of London, London, United Kingdom

**Keywords:** systemic chemotherapy, carboplatin, germ cell tumours, seminoma, dysgerminoma, de-escalating chemotherapy

## Abstract

Malignant germ cell tumours are a group of rare cancers whose incidence peaks in late adolescence and early adulthood. Dysgerminomas of the ovary and seminomas of the testis are analogous diseases, but seminomas have a 10-fold higher incidence. The two tumours are morphologically identical and are only differentiated by surrounding organ-specific tissue or testicular germ cell neoplasia *in situ*. They share genetic features including *KIT* and *RAS* mutations, amplification of chromosome 12p, and expression of pluripotency markers (NANOG (Nanog homeobox), OCT3/4 (Octamer-binding transcription factor 3/4), and SAL4 (Spalt-like trascription factor 4)). Both histologies are exquisitely sensitive to platinum chemotherapy, and the combination of bleomycin, etoposide, and cisplatin (BEP) yields survival rates greater than 90%. However, BEP causes significant, lifelong toxicity (cardiovascular, renal, respiratory, and neurological) in these young patients with an expectation of cure. Here, we comprehensively review the biological features of dysgerminoma and seminoma to demonstrate that they are biologically analogous diseases. We present available clinical trial data supporting de-escalation of chemotherapy treatment. Finally, we propose that future trials should enrol men, women, and children to benefit all patients regardless of age or sex.

## 1 Introduction

Approximately 90% of malignant tumours of the testis have a germ cell histology Testicular Germ Cell Tumour (TGCT). Seminomas are the most common pathological subtype accounting for 50% of testicular cancers worldwide (1). In contrast, malignant ovarian germ cell tumours (mOGCTs) are rare, accounting for less than 5% of ovarian malignancies (2, 3), but, of these, dysgerminomas represent 30%–40% (4). Dysgerminomas have a 10-fold lower incidence than seminomas and an earlier age of onset, peaking at 1.2 per 100,000 in women aged 15–19 (5), whereas seminomas have an incidence of 10.1 per 100,000 men aged 15–40 (6) and a mean age of 35 years at diagnosis (7). Both tumours are characterised by exquisite sensitivity to chemotherapy and an excellent prognosis, even when patients present with metastatic disease. Current treatment guidelines recommend widespread use of BEP (bleomycin, etoposide, and cisplatin) or EP (etoposide and cisplatin) chemotherapy with significant associated toxicity.

Here, we present evidence outlining the biological and clinical similarities between seminoma and dysgerminoma. We discuss recent clinical trials and our regional clinical experience, both of which strongly support a move towards de-escalation of systemic chemotherapy in adults in line with paediatric practice. We propose that future clinical trials should enrol patients with dysgerminoma and seminoma of all ages to increase statistical power, promote scientific discovery, and improve outcomes for all patients.

## 2 Biological features

### 2.1 Embryological origin and genetic features

TGCTs and mOGCTs have common developmental origins. Seminomas develop from premalignant germ cell neoplasia *in situ* (GCNIS) within seminiferous tubules of the testes (8), whereas dysgerminomas arise from primordial germ cells (PGCs) (9). Embryonic transcription factors such as NANOG and OCT4 promote proliferation and accumulation of mutations in PGCs (10). Immunohistochemical analysis has quantified an increase in NANOG and OCT4 expression of at least 1.5- fold compared with healthy testis in 33% and 56% of seminomas, respectively (11), whereas, in dysgerminomas, 67% exhibit positive staining for NANOG and 80% for OCT3/4 (12). In contrast, expression of these embryonic stem cell markers is minimal in other GCT histologies.

Seminomas and dysgerminomas also share genetic and epigenetic aberrations that are not found in other GCTs. Recurrent DNA copy number alterations have been identified in dysgerminoma, specifically gain of chromosomes 7, 8, 12, and 21 and loss of chromosome 13 (13). Although chromosome 12p accumulation is a common feature in many GCTs, it is considered a hallmark of seminoma and is also observed in up to 77% of mOGCTs (13, 14). Mutations in *KIT* and *RAS* proto-oncogenes are identified in approximately 30% of dysgerminoma and seminoma (13–15) with targeted sequencing revealing five mutations located in domains that are common to both seminoma and dysgerminoma (14). In seminoma, *KIT* mutation likely occurs pre-migration of PGC to the gonadal ridges, whereas these mutations arise post-migration in dysgerminoma. This difference is likely explained by sex-specific PGC differentiation into either oocytes or spermatozoa (13).

### 2.2 Morphology

**Figure 1:**
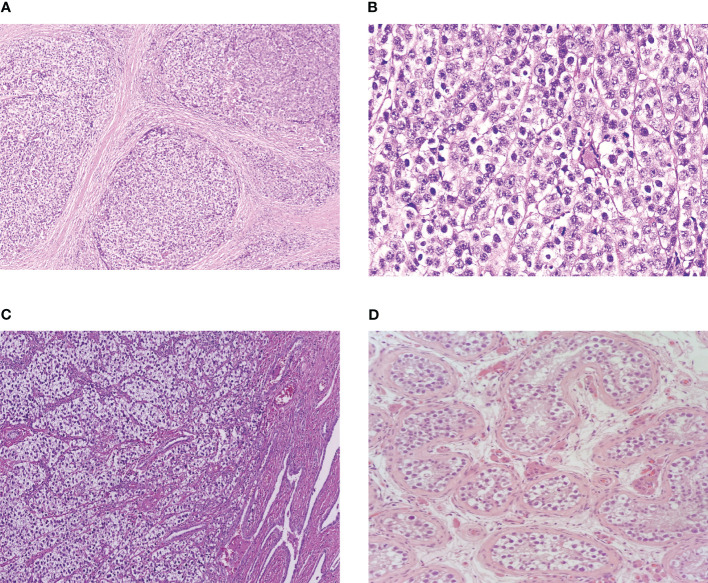
**(A)** Ovarian dysgerminoma at low power. **(B)** Ovarian dysgerminoma at high power. **(C)** Testicular seminoma invading rete testis. **(D)** Germ cell carcinoma neoplasia *in situ*.

Seminoma and dysgerminoma are morphologically indistinguishable (16, 17) and can only be differentiated by surrounding organ-specific tissue or testicular GCNIS (**Figure 1**). Both tumours are composed of uniform round primitive germ cells with clear cytoplasm and macronucleoli arranged in nests or cords and separated by thin fibrovascular septae containing lymphocytes (16, 17). Vascular invasion is less common than other GCTs but is estimated to be present in 34% of seminomas (17) and may have prognostic significance (18). Although data are more limited in dysgerminoma, vascular invasion is also uncommon and has been described in approximately 20% of cases (19). Immunohistochemical staining reflects the shared genetic basis of the two cancers, and positive staining for OCT3/4 and SALL4 as well as strong membrane positivity for c-KIT (CD117) and D2-40 are used clinically to define both seminoma and dysgerminoma (20, 21).

## 3 Treatment

### 3.1 General principles

In the UK, all patients with cancer are managed by diverse professional groups within multi-disciplinary teams (MDTs) based at high- volume, specialist oncology centres. For rare tumours like dysgerminomas and seminomas, “supra-regional” MDTs cover several hospitals and large geographical areas and include urological, gynaecological, and paediatric oncologists and surgeons. This specialised, collaborative approach has been shown to improve patient outcomes (22). Although surgery and radiotherapy for testicular, ovarian, and extra-gonadal mGCTs are necessarily different, owing to their different anatomical sites, there is a great deal of overlap in systemic chemotherapy.

### 3.2 Platinum-based chemotherapy

GCTs at all anatomical sites show excellent sensitivity to platinum. This has been attributed to the preference of germ cells to respond to DNA damage by promoting apoptosis, as opposed to DNA repair pathways, perhaps to reduce the likelihood of altering the germline (23). Even among GCTs, seminoma and dysgerminoma are both particularly sensitive to platinum chemotherapy, potentially implying that common mechanisms of drug response are shared between these two tumours. This is reflected in an overall survival (OS) of 96% and 89% for good and intermediate prognosis metastatic seminoma, respectively (24). In dysgerminoma, first- line treatment of FIGO (International Federation of Gynaecology and Obstetrics) stage IV disease results in a 5-year OS greater than 90% (25, 26) compared with 72% for stage IV mOGCTs overall. Interestingly, a correlation has been shown between DNA methylation and response of TGCTs (excluding teratomas) to cisplatin chemotherapy, and, because cytogenetic studies have identified global hypomethylation in pure seminoma and dysgerminoma relative to other GCTs (27, 28), this could contribute to their extreme platinum sensitivity.

### 3.3 International treatment guidelines

Current international guidelines for the treatment of mOGCTs in adults (National Comprehensive Cancer Network (29) and European Society of Medical Oncology) (3), children, and adolescents and young adults (AYA) (ESGO/SIOPE) (30) recommend that ovary confined, FIGO stage I pure dysgerminomas, which have been “properly surgically staged,” can be safely treated with surgery alone. Likewise, stage I seminomas can be treated with radical orchidectomy followed by surveillance without the need for adjuvant treatment (31) although discussion with patients regarding the marginal benefit of adjuvant carboplatin or radiotherapy to a para-aortic field is advised (32). In higher-stage dysgerminoma (FIGO stages II–IV), the guidelines recommend fertility sparing surgery followed by four cycles of BEP chemotherapy (omitting bleomycin from the fourth cycle). A similar treatment approach is recommended for adult men with metastatic seminoma, although patients presenting with IGCCCG (International Germ Cell Cancer Collaborative Group) good-prognosis metastatic disease receive three rather than four cycles of BEP or four cycles of EP (24, 33).

### 3.4 Second-line treatment

In the small number of patients who relapse, treatment is generally with combination chemotherapy, but there is no consensus on the optimal regimen. High-dose chemotherapy followed by autologous bone marrow transplant may also be used. Multiple novel targeted agents have been tested in GCTs. Although some activity has been observed with the cyclin dependent kinase 4/6 inhibitor palbociclib in patients with teratoma (34, 35), results from early phase trials testing other targeted agents such as angiogenesis inhibitors (36, 37), mammalian Target Of Rapamycin (mTOR) inhibitors (38, 39), and Programmed Death Ligand 1 (PDL1) inhibitors (40) have generally been poor. Even agents with a relevant target, for example, the c-KIT inhibitor imatinib in patients with c-KIT–expressing GCT (41), have not demonstrated efficacy.

### 3.5 Treatment-related toxicity

Although chemotherapy is highly effective, it is associated with profound adverse short- and long-term effects. For example, BEP can cause ototoxicity (20%–50% patients), pulmonary toxicity (1%–3% fatality), renal impairment (mean decrease in glomerular filtration rate 25.9% with ≥3 cycles), neurotoxicity (20%–40% patients), cardiovascular morbidity (7% angina and 25%–61% Raynaud’s phenomenon), and secondary malignancies (e.g., approximately two-fold increased risk of leukaemia in patients treated with combined BEP and radiotherapy) (42–45). Because the vast majority of patients with seminoma and dysgerminoma are young and will ultimately be cured of their disease, a focus on survivorship and quality of life following curative treatment is of utmost importance.

One successful way to achieve this in seminoma has been the use of radiotherapy in stage I–IIb disease, which has been shown to prevent disease recurrence following orchidectomy in 15%–20% of these patients (32). However, this approach is associated with increased risk of secondary malignancies (42, 45). Dysgerminomas are also radiosensitive, but anatomical considerations including the frequently large size of the primary tumour, together with the risk of secondary cancers and premature ovarian failure, preclude radiotherapy in women with this disease (46).

### 3.6 Carboplatin-based regimens

#### 3.6.1 Paediatric and AYA

In the absence of effective therapeutic alternatives to platinum, one approach to reducing chemotherapy toxicity, whilst maintaining anti-cancer efficacy has been to use carboplatin instead of cisplatin. Trials have focused on paediatric and AYA patients, perhaps because hearing loss in particular has significant educational, social, and vocational consequences in young patients (47). In pre-pubertal men and women up to age 18 with stage II–IV extracranial mGCTs, adjuvant chemotherapy with four cycles of JEB (bleomycin of 15 iu/m^2^ on day 1, etoposide of 120 mg/m^2^ on days 1–3, and carboplatin of 600 mg/m^2^ on day 1) has been used safely for over 20 years (48). Reported OS rates are more than 90% (49), which compares favourably with cisplatin-based regimens (50, 51), but treatment-related deafness and pulmonary and renal toxicity are extremely rare (49, 52).

In dysgerminoma, the most compelling evidence that carboplatin-based chemotherapy has equivalent efficacy to cisplatin in children and AYAs derives from a recent meta-analysis of paediatric and AYA patients treated in six international GCT trials including 126 patients with dysgerminoma (mean age, 20 years). There was no difference in Event-Free Survival (EFS) or OS between patients who were treated with cisplatin (n = 70) (5-year EFS rate, 93%; OS rate, 96%) and those who received carboplatin (n = 56) (5-year EFS rate, 96%; OS rate, 96%) (25). Another recent single-arm observational study of patients younger than 18 years, including 12 patients with dysgerminoma, demonstrated the safe use of JEB with an OS for the whole group of 97%, 5-year EFS of 92%, and no significant hearing or renal side effects (52).

#### 3.6.2 Adult seminoma and dysgerminoma

Evidence for carboplatin-based chemotherapy in the treatment of adult patients is much more sparse. In the 1990s, two clinical trials compared single-agent carboplatin to cisplatin-based chemotherapy in adults with good prognosis metastatic seminoma. The first trial randomised between four and six cycles of carboplatin (400 mg/m^2^) and four cycles of VIP (etoposide of 100 mg/m^2^, ifosfamide of 1,200 mg/m^2^, and cisplatin of 20 mg/m^2^ on days 1–5), with both being administered every 28 days. The second trial compared four cycles of carboplatin (400 mg/m^2^) to EP chemotherapy (etoposide of 120 mg/m^2^ on days 1–3 and cisplatin 20 mg/m^2^ days 1–5) administered every 21 days. Both trials demonstrated higher rates of treatment failure with single-agent carboplatin compared with the combination regimens (53). This could be due to the additional drugs used in the combination regimens, and the trials have since been criticised for using an inadequate dose and frequency of carboplatin.

In dysgerminoma, a small single-arm study of 39 paediatric and adult patients investigated adjuvant carboplatin and etoposide without bleomycin following complete surgical resection (54) (the paediatric and AYA cases from this study were included in the meta-analysis presented in Section 3.6.1 (25). Notably, this trial included 21 patients with stage II/III disease and only 11 patients older than 30 years. Despite using carboplatin (400 mg/m^2^) every 28 days, survival rate was still 100% after a median follow up of 7.8 years with acceptable toxicity (54). This trial was unfortunately terminated early because of non-favourable results in parallel studies in non-seminomatous GCTs (55, 56). Because no other clinical trials have been conducted, the role of carboplatin in adult dysgerminoma remains unresolved.

## 4 High-dose, single-agent carboplatin

To address the limitations of previous trials, we recently investigated the use of high-dose, single-agent carboplatin [area under the curve × 10 (AUC10) in accordance with the Calvert formula, every 3 weeks for four cycles] in 216 men with good-prognosis metastatic seminoma in a multi-centre phase II, non-randomised study. Efficacy was similar to BEP with a 5-year disease-specific OS of 98.3% (57). Importantly, we observed favourable toxicity in terms of febrile neutropenia and nephrotoxicity. We anticipate that long-term effects such as ototoxicity will be reduced although this remains to be determined.

These results lead us to adopt single-agent AUC10 as our preferred adjuvant treatment for good risk metastatic seminoma in our Supra-network Germ Cell Tumour MDT. On the basis of the similar embryological origins, morphologic, genetic, and clinical features of seminoma and dysgerminoma, we have extrapolated our use of carboplatin AUC10 to include ovarian dysgerminoma. This approach is subject to ongoing evaluation as a multi-centre, prospective, longitudinal study in the nine hospitals that make up our MDT. To promote further clinical investigation of this regimen, we now report the dysgerminoma cases that we have treated to date and have at least 3 years follow-up after completion of chemotherapy. The first patient who we treated with single-agent carboplatin was presented to our MDT in March 2017, and the last patient with sufficient follow-up was presented in July 2019. Between these two dates a total of eight patients with newly diagnosed dysgerminoma were discussed, and, because our MDT has a referral population of 7.5 million, this low case number highlights the very low incidence of this disease.

Two of the eight patients with dysgerminoma were FIGO stage I and were managed with post-operative observation. The other six all received single-agent carboplatin (**Table 1**). These six women have now been followed up for a median of 59.5 months (range, 36–73), and none have relapsed. Because most GCT recurrence occurs within 2 years of treatment (58), this implies that carboplatin AUC10 is sufficiently efficacious for dysgerminoma although we acknowledge that late relapse can occur and will only be detected by longer observation.

**Table 1:** Summary of clinical features.

Age at diagnosis	Presenting features	FIGO stage	Surgery	Adjuvant chemotherapy	Chemotherapy toxicities	Chemotherapy dose reduction/delays
16	RIF pain and complex adnexal mass	II	Right salpingo-oophrectomy and *en bloc* resection of pelvic peritoneum	Carboplatin AUC 10 × 3	G1 nausea, G1 fatigue, and G2 anaemia	–
25	RIF pain and suspected appendicitis	IIIA1(ii)	Laparoscopic right salpingo-oophrectomy and adhesiolysis	Carboplatin AUC 10 × 3	G1 nausea, G1 fatigue, and G4 thrombocytopenia	Cycle 3: Delay due to thrombocytopenia (G4)
23	Abdominal distension	IIIA(ii)	Left salpingo-oophrectomy, mesenteric lymphadenopathy, and para-aortic lymphadenopathy	Carboplatin AUC 10 × 3	G3 diarrhoea	–
58	RIF pain	IVB	Total abdominal hysterectomy, bilateral salpino-oophrectomy, appendicectomy, pelvic sidewall tumour resection, right pelvic node debulking, and adhesiolysis	Carboplatin AUC 10 × 3	G1 CIPN, G3 ototoxicity, G4 thrombocytopenia, and G4 neutropenia	Cycle 3: Dose reduction (20%) and delay due to thrombocytopenia (G4) and neutropenia (G4)
16	Primary amenorrhoea and pelvic mass	1C	Bilateral sapingo-oophrectomy, partial omentectomy, and debulking of para-aortic mass	Carboplatin AUC 10 × 4	G3 thrombocytopenia and G3 neutropenia	Cycle 3: Dose reduction (20%) and delay due to thrombocytopenia (G3) and neutropenia (G3)
16	Abdominal and back pain, and ovarian cyst	IIB	Right salpingo-oophrectomy, infracolic omentectomy, peritoneal excision biopsies from pouch of Douglas, and left peritoneal peritoneum	Carboplatin AUC 10 × 3	G2 vomiting, G3 anaemia, and G4 thrombocytopenia	Cycle 3: Dose reduction (20%) and delay due to thrombocytopenia (G4)

RIF, right iliac fossa; CIPN, chemotherapy-induced peripheral neuropathy; G, common toxicity criteria for adverse events, grade.

Carboplatin AUC10 was extremely well tolerated. Low-grade nausea and diarrhoea were common, occurring in four of the six patients. Bone marrow toxicity, particularly thrombocytopenia, was the most frequent grade 3/4 toxicity, occurring in four patients. All cases of haematological toxicity were readily managed with treatment delays and dose reductions and none developed neutropenic sepsis. We made a similar observation in men where 37% and 27% suffered grade 3/4 neutropenia and thrombocytopenia, respectively (57). Four of the six women were in the AYA age group, and none of these patients experienced long-term toxicity. This compares to our prior published data showing rates of chronic toxicity related to BEP in 20% of mOGCTs treated in our region (59).

It is of particular interest that efficacy was also seen in a 58-year-old patient with dysgerminoma, in whom BEP would have been contraindicated due to the high risk of pulmonary toxicity in patients older than 40 years (60, 61). This patient had poor risk disease due to the high stage (FIGO IVB) and the fact that age greater than 45 years is a known adverse risk factor in dysgerminoma (24, 62). Therefore, her relapse-free survival at 70 months follow-up is particularly encouraging. It is, however, important to note that this patient continues to have peripheral neuropathy and hearing impairment and so even carboplatin AUC10 should be used with caution in this patient age group, where GCTs are exceptionally rare.

Importantly, all six women remain in remission at a median of 5 years of follow-up. Our data, therefore, provides preliminary evidence of efficacy and tolerability of single-agent carboplatin AUC10 in metastatic dysgerminoma in line with our much larger metastatic seminoma cohort (n = 216) (57). In light of these new data, we now recommend investigation of this regimen in a larger, randomised clinical trial enrolling seminoma and dysgerminoma with the aim of reducing treatment-related toxicities for all patients.

## 5 Conclusions

The existing literature presented here demonstrates that dysgerminomas biologically resemble seminomas more closely than any other GCTs. In the 2022 World Health Organisation classification of genitourinary tumours, seminoma is now placed in the “germinoma family” of tumours (63), emphasising that dysgerminoma, seminoma, and germinoma are the same tumour arising at different sites. This similarity is borne out in clinical practice by comparable efficacy of systemic treatments.

We have now implemented carboplatin AUC10 as standard treatment for good prognosis metastatic seminoma in our region and single-agent carboplatin is already being used more widely across the UK in good prognosis disease (64). The new data that we present here show, for the first time, that women with dysgerminoma can also be successfully treated with carboplatin AUC10 with minimal associated toxicity. We are now prospectively collecting data for all patients with dysgerminoma and seminoma receiving this regimen as an ongoing multi-centre cohort study.

The rarity of dysgerminoma has resulted in a paucity of suitable clinical studies; thus, treatment advances for patients with dysgerminoma have fallen behind those for men with seminoma. This situation is highlighted by the fact that, at the time of writing, there are only seven clinical trials for patients with dysgerminoma listed on clinicaltrials.gov, of which only one is recruiting patients. This compares to 59 clinical trials for seminoma. In view of the notable similarities between dysgerminoma and seminoma, we propose that they should be investigated together in the same clinical trials. Such a commitment requires international collaboration, particularly in rare cancers. The ongoing AGCT1531 trial (65) for non-seminoma/dysgerminoma GCTs that will enrol patients regardless of patient age or sex demonstrates the feasibility of conducting clinical trials that transcend established professional boundaries. Future investigators should build on this approach by developing similarly inclusive trials to facilitate equitable improvements in outcome for all patients with these rare cancers.

## Data availability statement

The original contributions presented in the study are included in the article/supplementary material. Further inquiries can be directed to the corresponding author.

## Ethics statement

Ethical approval was not required for the study involving humans in accordance with the local legislation and institutional requirements. Written informed consent to participate in this study was not required from the participants or the participants’ legal guardians/next of kin in accordance with the national legislation and the institutional requirements.

## Author contributions

GW: Data curation, Formal Analysis, Investigation, Writing – original draft, Writing – review & editing. CB: Data curation, Formal Analysis, Investigation, Writing – original draft, Writing – review & editing. MV: Data curation, Formal Analysis, Investigation, Writing – original draft, Writing – review & editing. RA: Data curation, Formal Analysis, Visualization, Writing – review & editing. DB: Data curation, Formal Analysis, Visualization, Writing – review & editing. CA: Conceptualization, Data curation, Writing – review & editing. NM: Conceptualization, Data curation, Writing – review & editing. RM: Conceptualization, Data curation, Formal Analysis, Investigation, Methodology, Supervision, Writing – review & editing. JS: Conceptualization, Data curation, Formal Analysis, Investigation, Methodology, Supervision, Writing – review & editing. SS: Conceptualization, Data curation, Formal Analysis, Investigation, Methodology, Supervision, Writing – review & editing. ML: Conceptualization, Data curation, Formal Analysis, Investigation, Methodology, Project administration, Supervision, Validation, Visualization, Writing – original draft, Writing – review & editing.
